# Supplementary material for: Splice-Junction-Based Mapping of Alternative Isoforms in the Human Proteome
Source: Cell Rep. Author manuscript; Available in PMC 2020 Jan 15. (PMC6961840; doi:10.1016/j.celrep.2019.11.026)
Supplement: 2 [file NIHMS1546469-supplement-2.zip › DF1/PXD010154/Heart/Heart_68_AAK1_TAAEDSNLISGFDVPEGSDKVAEDEFDPIPVLITK.pdf]

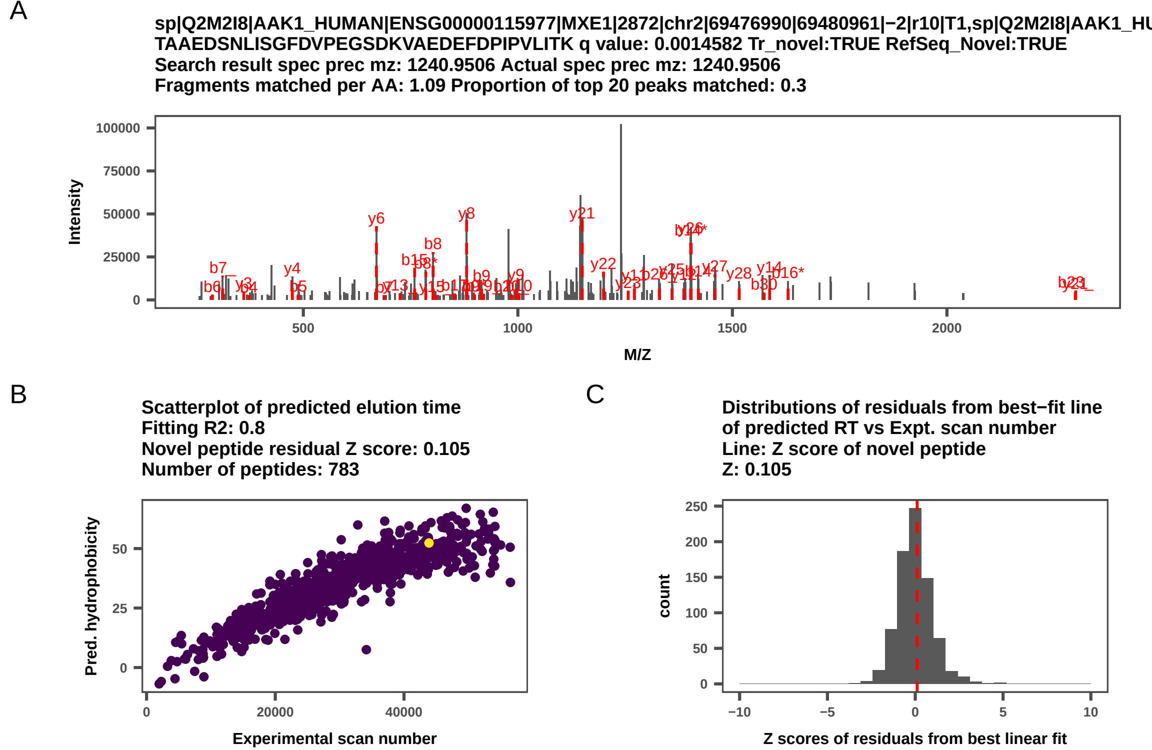
A
sp|Q2M2I8|AAK1_HUMAN|ENSG00000115977|MXE1|2872|chr2|69476990|69480961|−2|r10|T1,sp|Q2M2I8|AAK1_HU
TAAEDSNLISGFDVPEGSDKVAEDEFDPIPVLITK q value: 0.0014582 Tr_novel:TRUE RefSeq_Novel:TRUE
Search result spec prec mz: 1240.9506 Actual spec prec mz: 1240.9506
Fragments matched per AA: 1.09 Proportion of top 20 peaks matched: 0.3
Intensity
100000
75000
50000
25000
0
y6
y8
y21
b8
y22
b7_
y4
b5
b6
b9
y9
y13
y27
y28
y14
b16*
b30
500
1000
1500
2000
M/Z
B
Scatterplot of predicted elution time
Fitting R2: 0.8
Novel peptide residual Z score: 0.105
Number of peptides: 783
Pred. hydrophobicity
50
25
0
20000
40000
Experimental scan number
C
Distributions of residuals from best−fit line
of predicted RT vs Expt. scan number
Line: Z score of novel peptide
Z: 0.105
count
250
200
150
100
50
0
−10
−5
5
10
Z scores of residuals from best linear fit
